# Disparities in Oral Cancer Survival among Mentally Ill Patients

**DOI:** 10.1371/journal.pone.0070883

**Published:** 2013-08-07

**Authors:** Ting-Shou Chang, Szu-Jen Hou, Yu-Chieh Su, Li-Fu Chen, Hsu-Chieh Ho, Moon-Sing Lee, Chun-Hsuan Lin, Pesus Chou, Ching-Chih Lee

**Affiliations:** 1 Department of Otolaryngology, Kaohsiung Veterans General Hospital, Kaohsiung, Taiwan; 2 Department of Otolaryngology, Buddhist Dalin Tzu Chi General Hospital, Chiayi, Taiwan; 3 Cancer Center, Buddhist Dalin Tzu Chi General Hospital, Chiayi, Taiwan; 4 Department of Radiation Oncology, Buddhist Dalin Tzu Chi General Hospital, Chiayi, Taiwan; 5 Division of Hematology-Oncology, Department of Internal Medicine, Buddhist Dalin Tzu Chi General Hospital, Chiayi, Taiwan; 6 School of Medicine, Tzu Chi University, Hualian, Taiwan; 7 Department of Medical Research, Buddhist Dalin Tzu Chi General Hospital, Chiayi, Taiwan; 8 Department of Emergency, National Yang-Ming University Hospital, Taipei, Taiwan; 9 Community Medicine Research Center and the Institute of Public Health, National Yang-Ming University, Taipei, Taiwan; University of Wisconsin School of Medicine and Public Health, United States of America

## Abstract

**Background:**

Many studies have reported excess cancer mortality in patients with mental illness. However, scant studies evaluated the differences in cancer treatment and its impact on survival rates among mentally ill patients. Oral cancer is one of the ten most common cancers in the world. We investigated differences in treatment type and survival rates between oral cancer patients with mental illness and without mental illness.

**Methods:**

Using the National Health Insurance (NHI) database, we compared the type of treatment and survival rates in 16687 oral cancer patients from 2002 to 2006. The utilization rate of surgery for oral cancer was compared between patients with mental illness and without mental illness using logistic regression. The Cox proportional hazards model was used for survival analysis.

**Results:**

Oral cancer patients with mental disorder conferred a grave prognosis, compared with patients without mental illness (hazard ratios [HR] = 1.58; 95% confidence interval [CI] = 1.30–1.93; *P*<0.001). After adjusting for patients’ characteristics and hospital characteristics, patients with mental illness were less likely to receive surgery with or without adjuvant therapy (odds ratio [OR] = 0.47; 95% CI = 0.34–0.65; *P*<0.001). In multivariate analysis, oral cancer patients with mental illness carried a 1.58-times risk of death (95% CI = 1.30–1.93; *P*<0.001).

**Conclusions:**

Oral cancer patients with mental illness were less likely to undergo surgery with or without adjuvant therapy than those without mental illness. Patients with mental illness have a poor prognosis compared to those without mental illness. To reduce disparities in physical health, public health strategies and welfare policies must continue to focus on this vulnerable group.

## Introduction

Oral cancer is among the ten most common forms of cancer in the world [1]. A trend of rising incidence has been noted on a global scale in Western countries as well as Asian countries such as Taiwan [2], [3]. Among all cancers in Taiwanese males, oral cancer which contributed 70% of head and neck cancer has been ranked fourth in incidence and mortality since 1995. It is an obvious consequence that the treatment of oral cancer makes an increasing economic burden [4], [5].

Previous reports revealed excess cancer mortality in psychiatric patients, and several possible mechanisms were proposed [6]–[8]. Patients with mental illness are associated with medical comorbidity, unemployment, living alone and low socioeconomic status [9], [10]. Due to the problems of cognitive, affective, and social manifestations, it may be hard to get complete informed consent from mentally ill patients [11]. Reduced access to general medical care and physician discretion for ordering examination may decrease the probability of full evaluation of patients with mental illness [7], [12]. High incidence of postoperative complications in patients with mental illness may further compromise the survival rates [13]. However, there is no large-scale studies which explored the receipt of medical care and survival rate among oral cancer patients with mental illness.

Although the implementation of the National Health Insurance in Taiwan in 1995 may reduce the financial barriers to access medical care for all diseases, there may be some disparities in treatment modality and long-term survival for oral cancer between patients with and without mental illness. We hypothesize here that oral cancer patients with mental illness tend to have higher risk of mortality, compared with those without mental illness. To address this hypothesis, we evaluate the association between mental illness and 5-year survival rates among oral cancer patients through the National Health Insurance Research Database (NHIRD) in Taiwan. Using a population-based data allows us to trace all medical service utilization history among oral cancer patients and measure the relationship of mental illness with oral cancer survival rates.

## Materials and Methods

### Ethics Statement

This study was approved by the Institutional Review Board of Buddhist Dalin Tzu Chi General Hospital, Taiwan. Review board requirements for written informed consent were waived because all personal identifying information was removed from the dataset prior to analysis.

### Database

The data for this study were collected from Taiwan’s NHIRD for the years 2002 to 2006. This dataset is organized and managed by Taiwan’s National Health Research Institutes but collected by Taiwan’s National Health Insurance Program, which has been in place in Taiwan since 1995. The program covers approximately 99% of the residents in Taiwan and has contracts with 97% of the medical providers there [14]. To verify accuracy of diagnoses, Taiwan’s Bureau of National Health Insurance randomly reviews the charts of one per 100 ambulatory and one per 20 inpatient claims [15], [16].

Our cohort study consisted of Taiwan’s incidental oral cancer patients (*International Classification of Diseases, Ninth Revision, Clinical Modification* [ICD-9-CM] codes 140.0–145.9; malignant salivary gland tumor [ICD code 142] was excluded) who had undergone treatment from 2002 and 2006. A total of 16687 patients with oral cancer with treatment were identified. In addition, cancer staging was not known in our database and neither was smoking status.

### Independent Variables

Admission ICD9-CM diagnosis identified patients with or without coexisting mental illness deemed current and ongoing at the time of initial diagnosis: (1) no mental illness, and (2) mental illness with a diagnosis of schizophrenia (ICD9-CM codes 295.00–295.99) or major affective disorder (ICD9-CM codes 296.00–296.99), or other major mental illness (ICD9-CM codes 290.00–294.99, 297.00–319.99).

Other patient characteristics contained age, gender, and severity of comorbid disease, socioeconomic status, geographic region, and urbanization of residence. The NHIRD didn’t have information on cancer staging. The disease severity of each patient was based on the Charlson Comorbidity Index score, which is widely used in recent years for risk adjustment in administrative claims data sets. We used a modified Charlson Comorbidity Index score, which is calculated as the sum of weighted scores based on the relative mortality risk of 19 conditions [17]. This study used labor enrollee category (EC) as a proxy measure of socioeconomic status (SES), which is an important prognostic factor for cancer [18], [19]. The oral cancer patients were classified into four groups: EC1 (civil servants, full-time, or regularly paid personnel with a government affiliation), EC2 (employees of privately owned institutions), EC3 (self-employed individuals, other employees, and members of the farmers’ or fishermen’s association), and EC4 (veterans, jobless families, and substitute service draftees) [20]. These patients were then further classified into three subgroups: EC1–2 (high SES), EC3 (moderate SES), and EC4 (low SES).

Geographical regions and urbanization were included. The level of urbanization was determined by population density, percentage of residents with college or higher education, percentage of residents over 65 years of age, percentage of residents who were agriculture workers, and the number of physicians per 100,000 people. The level of urbanization was assigned as urban, suburban, and rural areas [21].

The hospitals were categorized by ownership (public, non-for-profit or for-profit), and hospital accreditation level (medical center, regional or district hospital).

### Dependent Variables

The key dependent variable of interest was the 5-year survival of the patients. Rates of undergoing treatment were also studied.

### Statistical Analysis

The SAS statistical package (version 9.2; SAS Institute, Inc., Cary, N.C.) and SPSS (version 15, SPSS Inc., Chicago, IL, USA) were used to analyze this data. A two-sided value of *P*<0.05 was used to determine statistical significance.

Pearson’s chi-square tests were used to explore the differences between categorical variables. The cumulative 5-year survival rates and the survival curves were constructed with Kaplan-Meier method and compared by the log-rank test. Survival was measured from the time of the oral cancer diagnosed by using overall mortality as event variables. These patients were tracked for 5 years and further linked to the administrative data for the period 2002–2006 to estimate overall survival, with cases censored for patients who drew back guarantees from the National Health Insurance Program or were still robust without defined events at end of follow-up. A multiple logistic regression model was constructed to estimate adjusted odds ratios for differences of treatment type between oral cancer patients with and without mental illness. The Cox proportional regression model was used to evaluate the effect of mental illness on survival rates after adjusting patients’ characteristics (age, gender, Charlson Comorbidity Index Score, urbanization, area of residence and enrollee category), treatment modality (surgery with or without adjuvant therapy, and radiotherapy/chemotherapy/chemoradiotherapy) and hospital characteristics (ownership and accreditation level).

## Results

Of 16687 patients, 206 (1.2%) had a secondary diagnosis of a mental illness; 174(84%) patients with other major mental disorders and 32(16%) patients with schizophrenia or other major affective disorders. The median follow-up time was 18.1 months. Table 1 shows the characteristics of the study sample according to the presence or absence of mental illness in oral cancer patients. Patients with mental illness were more likely to be younger and high socioeconomic status (*P*<0.001, and = 0.002; respectively). Oral cancer patients with mental illness were significantly less likely to be admitted to medical center (*P* = 0.004).

**Table 1:** Characteristics of oral cancer, 2002–2006 (*n* = 16687).

Characteristics	With mental illness (*n* = 206)		Without mental illness (*n* = 16481)		*P* value
	N	%	N	%	
***Patient characteristics***					
Age					<0.001
≦40	49	23.8	2429	14.7	
41–50	76	36.9	5253	31.9	
51–60	46	22.3	4686	28.4	
61–70	20	9.7	2749	16.7	
≧71	15	7.3	1364	8.3	
Gender					
Male	184	89.3	15149	91.9	0.175
Female	22	10.7	1332	8.1	
Charlson Comorbidity Index Score					0.342
≦3	146	70.9	12164	73.8	
>3	60	29.1	4317	26.2	
Geographic region					0.322
Northern	62	30.1	5432	33.0	
Central	48	23.3	3657	22.1	
Southern	83	40.3	6741	40.9	
Eastern	13	6.3	651	4.0	
Urbanization level					0.207
Urban	36	17.5	3722	22.6	
Suburban	97	47.1	7433	45.1	
Rural	73	35.4	5326	32.3	
Enrollee category					0.002
1–2	76	36.9	4450	27.0	
3	93	45.1	7821	47.5	
4	37	18.0	4210	25.5	
***Hospital characteristics***					
Hospital ownership					0.296
Public	56	27.2	4786	29.0	
Non-for-profit	98	47.6	8266	50.2	
For-profit	52	25.5	3429	20.8	
Hospital accreditation level					0.004
Medical center	146	70.9	13154	79.8	
Regional hospital	56	27.2	3004	18.2	
District hospital	4	1.9	323	2.0	

Values are given as number (percentage).

**Figure 1:**
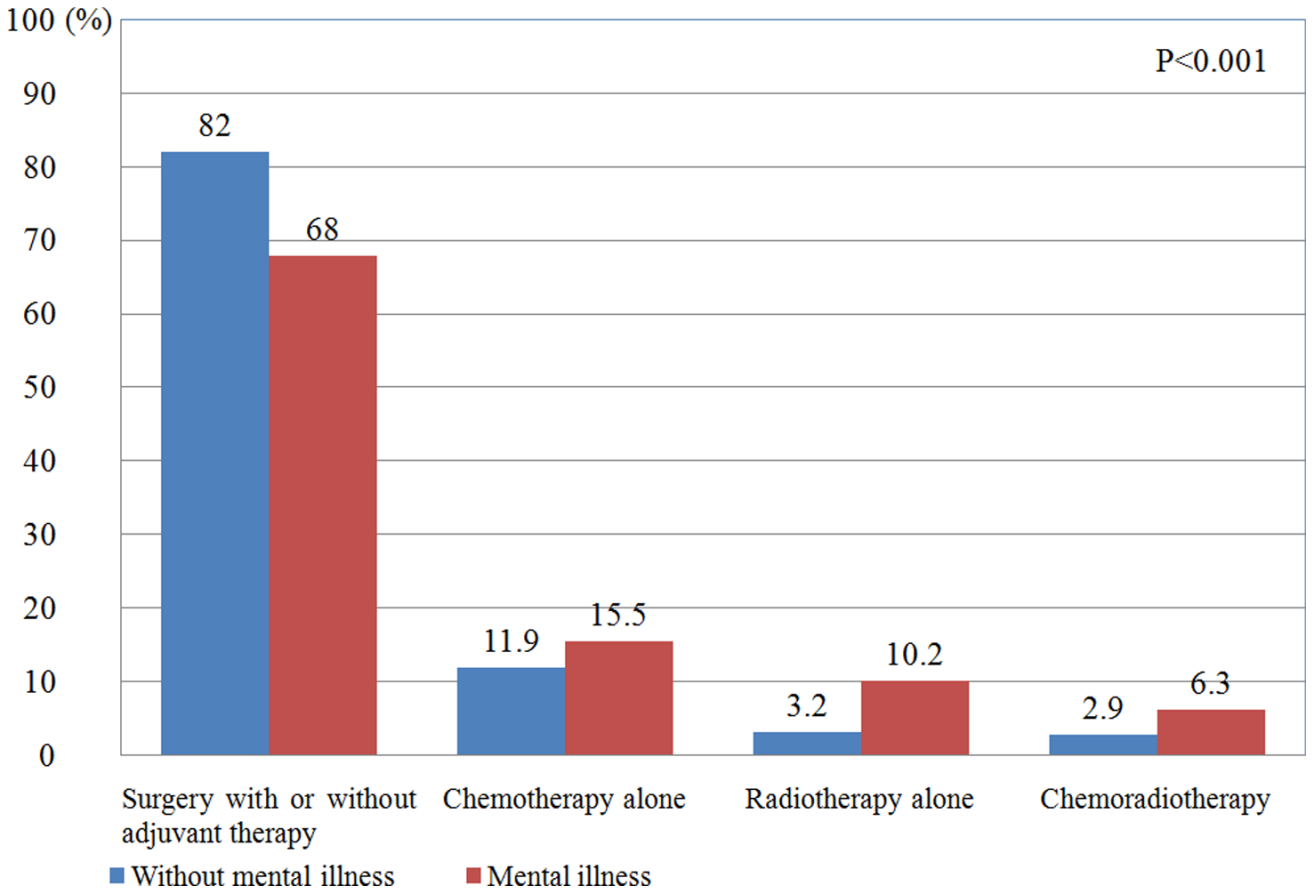
With or without mental illness in oral cancer patients for the treatment modality.

Figure 1 depicts the distribution of treatment modality between the two groups. After adjusting for age and gender, patients with mental illness were substantially less likely to undergo surgery with or without adjuvant therapy (odds ratio [OR] = 0.45; 95% confidence interval [CI] = 0.33–0.60; *P*<0.001; Table 2). After adjusting patients’ characteristics (age, gender, Charlson Comorbidity Index Score, urbanization, area of residence and enrollee category) and hospital characteristics (ownership and accreditation level), patients with mental illness remained less likely to receive surgery with or without adjuvant therapy (OR = 0.47; 95% CI = 0.34–0.65; *P*<0.001).

**Table 2:** Use of treatment in individuals with and without mental illness (*n* = 16687).

Characteristics	Model A			Model B*			Model C**		
	OR	95% CI	*P* value	OR	95% CI	*P* value	OR	95% CI	*P* value
Surgery with or without adjuvant therapy									
Without mental illness	1			1			1		
Mental illness	0.47	0.35–0.63	<0.001	0.45	0.33–0.60	<0.001	0.47	0.34–0.65	<0.001
Age				0.99			0.99	0.98–0.99	<0.001
Gender									
Male				1			1		
Female				1.63	1.39–1.93	<0.001	1.61	1.35–1.91	<0.001
Charlson Comorbidity Index Score							0.52	0.48–0.57	<0.001
Geographic region									
Northern							1		
Central							1.27	1.11–1.45	0.001
Southern							0.92	0.82–1.03	0.162
Eastern							0.59	0.47–0.73	<0.001
Urbanization level									
Urban							1		
Suburban							0.93	0.83–1.04	0.214
Rural							0.88	0.78–1.02	0.081
Enrollee category									
1–2							1		
3							1.06	0.95–1.19	0.291
4							0.75	0.67–0.85	<0.001
***Hospital characteristics***									
Hospital ownership									
Public							1		
Non-for-profit							0.82	0.73–0.92	0.001
For-Profit							0.19	0.17–0.22	<0.001
Hospital accreditation level									
Medical center							1		
Regional hospital							0.80	0.72–0.89	<0.001
District hospital							0.21	0.16–0.27	<0.001

95% CI, 95% confidence interval.

* Adjusted for patient age, and gender,

** Adjusted for patient age, gender, Charlson Comorbidity Index Score, geographic region, urbanization level, enrollee category, hospital ownership, and accreditation level.

**Figure 2:**
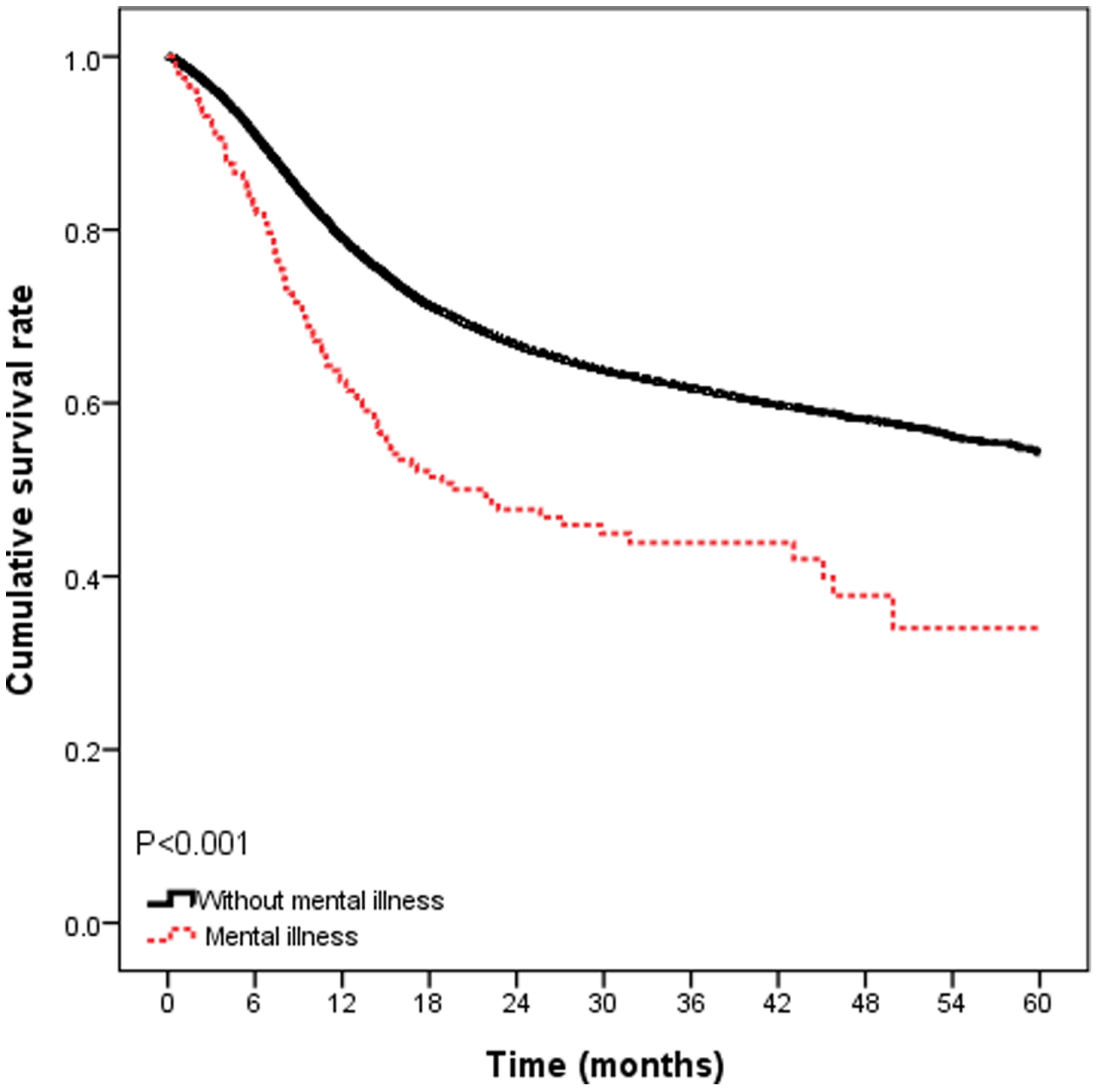
Survival rates in oral cancer patients with or without mental illness (*n* = 16687).

The 5-year survival rates, by presence or absence of mental illness, were illustrated in Figure 2. The 5-year survival rates were 50.5%, and 68.1% for patients with mental illness and without mental illness (*P*<0.001). In Cox proportional hazards models adjusted for patients’ characteristics and hospital characteristics, patients with mental illness conferred a significant increased risk for death (hazard ratios [HR] = 1.83; 95% CI, 1.50–2.23; *P*<0.001; Table 3). In models that additionally adjusted for treatment modality, patients with mental illness remained conferred a 1.58 times risk of death, compared to those without mental illness (HR = 1.58; 95% CI, 1.30–1.93; *P*<0.001).

**Table 3:** Hazard ratios for 5-year overall mortality in oral cancer patients with and without mental illness (*n* = 16687).

Characteristics	Model A			Model B*			Model C**		
	HR	(95% CI)	*P* value	HR	(95% CI)	*P* value	HR	(95% CI)	*P* value
Oral cancer patients									
Without mental illness	1			1			1		
Mental illness	1.86	1.53–2.27	<0.001	1.83	1.50–2.23	<0.001	1.58	1.30–1.93	<0.001
Age				1.01	1.01–1.01	<0.001	1.01	1.00–1.01	<0.001
Gender									
Male				1			1		
Female				0.82	0.73–0.91	<0.001	0.87	0.79–0.97	0.013
Charlson Comorbidity Index Score				2.26	2.14–2.39	<0.001	2.00	1.89–2.12	<0.001
Geographic region									
Northern				1			1		
Central				1.00	0.92–1.08	0.930	1.01	0.93–1.10	0.76
Southern				0.92	0.85–0.99	0.019	0.89	0.83–0.96	0.002
Eastern				1.10	0.95–1.28	0.197	0.99	0.85–1.15	0.884
Urbanization level									
Urban				1			1		
Suburban				1.05	0.97–1.13	0.224	1.05	0.97–1.13	0.227
Rural				1.14	1.05–1.25	0.002	1.15	1.06–1.25	0.001
Enrollee category									
1–2				1			1		
3				1.10	1.02–1.83	0.013	1.13	1.05–1.21	0.002
4				1.43	1.32–1.54	<0.001	1.38	1.28–1.49	<0.001
***Hospital characteristics***									
Hospital ownership									
Public				1			1		
Non-for-profit				1.00	0.93–1.07	0.979	0.97	0.91–1.04	0.40
For-profit				1.43	1.32–1.54	<0.001	1.07	0.98–1.16	0.13
Hospital accreditation level							2.68	2.52–2.85	
Medical center				1			1		
Regional hospital				1.23	1.14–1.32	<0.001	1.20	1.12–1.29	<0.001
District hospital				0.99	0.82–1.19	0.887	0.70	0.58–0.85	<0.001
Treatment modality									
Surgery with or without adjuvant therapy							1		
Radiotherapy/Chemotherapy/Chemoradiotherapy							2.68	2.52–2.85	<0.001

95% CI, 95% confidence interval.

* Adjusted for patient age, gender, Charlson Comorbidity Index Score, geographic region, urbanization level, enrollee category, hospital ownership, and accreditation level.

** Adjusted for patient age, gender, Charlson Comorbidity Index Score, geographic region, urbanization level, enrollee category, hospital ownership, accreditation level, and treatment modality.

## Discussion

This population-based study found that oral cancer patients with mental illness had a grave prognosis. Oral cancer patients with mental illness conferred a 1.58-times risk of mortality, compared with those without mental illness after adjusting for patient characteristics (age, gender, Charlson Comorbidity Index Score, urbanization, area of residence and enrollee category), hospital characteristics (ownership and accreditation level), and treatment modality. Patients with mental illness were substantially less likely to undergo surgery with or without adjuvant therapy. There was no significant difference in utilization of radiotherapy or chemotherapy or chemoradiotherapy in oral cancer patients with or without mental illness.

There are several possible mechanisms for poor prognosis in oral cancer patients with mental illness. Previous studies have explored that patients with mental illness were medically sicker and older, compared with patients without mental illness. Besides, oral cancer patients with severe comorbidity were associated with poor prognosis [9], [22]. In our study, mentally ill patients were more likely to be younger and from higher socioeconomic status. There was no significant difference in comorbidity severity between patients with mental illness and those without mental illness. Nevertheless, oral cancer patients with mental illness were prone to visit regional and district hospitals, which may lack the adequate facility, such as experienced head and neck surgeons, plastic surgeons, intensive care team, radiation oncologist, hematology oncologist and linear accelerator to treat oral cancer.

Oral cancer patients with mental illness were less likely to receive surgery with or without adjuvant therapy and the differences in rates of receipt of medical care between the two groups remained statistically significant in multivariate analyses that adjusted for patient characteristics, comorbidity score and hospital characteristics. Druss et al. used a large national survey in United States found that patients with mental illness tended to face particular difficulties in obtaining and maintaining both health insurance and needed medical care [12]. Although financial barriers to access medical care had been reduced after the implementation of the National Health Insurance in Taiwan in 1995, medical treatment modality remained different in mentally ill patients with either oral cancer or other disease [23]. This disparity should be of concern. Moreover, mentally ill patients are more likely to undergo postoperative complications, such as deep vein thrombosis, sepsis and respiratory failure [24]. These complications may delay the beginning of adjuvant radiotherapy or chemotherapy and cause poor prognosis for survival rates [25]. Furthermore, deteriorating psychiatric status was found more frequently in patients with mental disease [26]. Postoperative confusion, for example, may make the postoperative care more complicated and decrease the physicians’ intention to perform the surgery for oral cancer patients with mental illness.

Physician slant may exist when dealing with oral cancer patients with mental illness. One study demonstrated potential racial bias in influencing the decision of physicians to refer patients for cardiac catheterization [27]. This phenomenon may be observed among patients with mental illness in physical care [28], [29]. All the above reports indicated that potential physician bias may contribute to the difference of surgery rates in oral cancer treatment.

The reasons for excess mortality in oral cancer patients with mental illness may be associated with advanced disease and poor treatment compliance with therapy. Cognitive deficits, disorganized thinking, and impaired ability to communicate important medical symptoms may contribute to presentation at a later stage of disease [6], [30]. However, this phenomenon can’t be validated in our series due to lack of cancer staging in National Health Insurance Dataset. As regards interruption of the treatment course, such as radiotherapy or chemotherapy, may lower survival rates among mentally ill patients. Patients who had major deficiencies in treatment plans incurred a doubled risk of mortality compared with those who initially complied with treatment protocol [31], [32].

This study adds several novel points on the receipt of medical care and survival rates in oral cancer patients with or without mental illness. Using a population-based dataset enables us to trace all medical claims during the 5-year follow-up period. Previous reports presented the excess cancer mortality in psychiatric patients. However, the difference of medical care was not analyzed during these studies [6], [7]. Our study revealed the decreased access to medical treatment, especially surgery with or without adjuvant therapy among oral cancer patients with mental illness. This partly explained the poor survival rate in mentally ill patients. However, significant risk of death in patients with mental illness remained unaccounted for even in our fully adjusted model. Some unobservable variables, such as social isolation, and lack of family support may compromise the survival rates in patients with mental illness.

There are several limitations in this study. First of all, it is lack of access to detailed information from the insurance claims database with regard to oral cancer stage and pattern of relapse. These may be the important variables for increased mortality rate among cancer patients with mental illness. Delayed diagnosis or lack of access to screen in mentally ill patients might lead to more advanced staging at diagnosis and reduce possibility of surgical intervention after diagnosis. Further study is indicated using cancer registry data with more details on staging. Second, the database lacks information of lifestyle factors such as dietary habits, alcohol or tobacco use, which may be risk factors and prognostic factors for oral cancer [33]. Third, instead of cancer-specific survival rate, the overall survival rate was used, because it was not possible to determine cause-specific mortality based on this registry data. Mentally ill patients often have medical comorbidities and the direct cause of death may be another disease which susceptible to malignant neoplasm. Fourth, postoperative complication or treatment-related complications were not included. Fifth, the detail receipts of anti-psychotic drugs were not available. Given the magnitude and statistical significance of the observed effects in this study, these limitations are unlikely to compromise the results.

In summary, oral cancer patients with mental illness were substantially less likely to undergo surgery with or without adjuvant therapy than those without mental illness. Patients with mental illness conferred a poor prognosis in multivariate analysis. Oral cancer treatment for the mentally ill patients is a complex issue and deserves more concern. Meanwhile, there should be efforts to reduce disparities in physical health including oral mucosal screening during routine dental evaluations or physical examination.

## References

[1] Parkin DM, Bray F, Ferlay J, Pisani P (2005) Global Cancer Statistics, 2002. CA Cancer J Clin 55: 74–108. 15761078 10.3322/canjclin.55.2.74

[2] Mignogna MD, Fedele S, Russo LL (2004) The World Cancer Report and the burden of oral cancer. European Journal of Cancer Prevention 13: 139–142. 15100581 10.1097/00008469-200404000-00008

[3] Yang YH, Chen CH, Chang JS, Lin CC, Cheng TC, et al (2005) Incidence rates of oral cancer and oral pre-cancerous lesions in a 6-year follow-up study of a Taiwanese aboriginal community. J Oral Pathol Med 34: 596–601. 16202079 10.1111/j.1600-0714.2005.00266.x

[4] Lee J, Turini M, Botteman M, Stephens J, Pashos C (2004) Economic burden of head and neck cancer. The European Journal of Health Economics 5: 70–80. 15452768 10.1007/s10198-003-0204-3

[5] Lang K, Menzin J, Earle CC, Jacobson J, Hsu M-A (2004) The Economic Cost of Squamous Cell Cancer of the Head and Neck: Findings From Linked SEER-Medicare Data. Arch Otolaryngol Head Neck Surg 130: 1269–1275. 15545580 10.1001/archotol.130.11.1269

[6] Lawrence D, D’Arcy C, Holman J, Jablensky AV, Threfall TJ, et al (2000) Excess cancer mortality in Western Australian psychiatric patients due to higher case fatality rates. Acta Psychiatrica Scandinavica 101: 382–388. 10823298 10.1034/j.1600-0447.2000.101005382.x

[7] Kisely S, Sadek J, MacKenzie A, Lawrence D, Campbell LA (2008) Excess Cancer Mortality in Psychiatric Patients. La mortalité par cancer excessive chez les patients psychiatriques 53: 753–761. 10.1177/070674370805301107 19087469

[8] Tran E, Rouillon F, Loze J-Y, Casadebaig F, Philippe A, et al (2009) Cancer mortality in patients with schizophrenia. Cancer 115: 3555–3562. 19548261 10.1002/cncr.24383

[9] Jeste DV, Gladsjo JA, Lindamer LA, Lacro JP (1996) Medical Comorbidity in Schizophrenia. Schizophr Bull 22: 413–430. 8873293 10.1093/schbul/22.3.413

[10] Agerbo E, Byrne M, Eaton WW, Mortensen PB (2004) Marital and Labor Market Status in the Long Run in Schizophrenia. Arch Gen Psychiatry 61: 28–33. 14706941 10.1001/archpsyc.61.1.28

[11] Druss BG, Bradford DW, Rosenheck RA, Radford MJ, Krumholz HM (2000) Mental disorders and use of cardiovascular procedures after myocardial infarction. JAMA 283: 506–511. 10659877 10.1001/jama.283.4.506

[12] Druss BG, Rosenheck RA (1998) Mental Disorders and Access to Medical Care in the United States. Am J Psychiatry 155: 1775–1777. 9842793 10.1176/ajp.155.12.1775

[13] Copeland LA, Zeber JE, Pugh MJ, Mortensen EM, Restrepo MI, et al (2008) Postoperative complications in the seriously mentally ill: a systematic review of the literature. Ann Surg 248: 31–38. 18580204 10.1097/SLA.0b013e3181724f25

[14] NHI profile (2008) Available: http://www.nhi.gov.tw/english/webdata.asp?menu=11&menu_id=290&webdata_id=1884.Accessed 2008 June 15.

[15] Tseng CH (2004) Mortality and causes of death in a national sample of diabetic patients in Taiwan. Diabetes Care 27: 1605–1609. 15220235 10.2337/diacare.27.7.1605

[16] Bureau of National Health Insurance (2006) Available: http://www.nhi.gov.tw/information/bulletin_file/421_0890036465-19.doc.Accessed 2006 May 2.

[17] Deyo RA, Cherkin DC, Ciol MA (1992) Adapting a clinical comorbidity index for use with ICD-9-CM administrative databases. Journal of Clinical Epidemiology 45: 613–619. 1607900 10.1016/0895-4356(92)90133-8

[18] Braaten T, Weiderpass E, Lund E (2009) Socioeconomic differences in cancer survival: the Norwegian Women and Cancer Study. BMC Public Health 9: 178. 19505303 10.1186/1471-2458-9-178 PMC2702382

[19] Kwok J, Langevin SM, Argiris A, Grandis JR, Gooding WE, et al (2010) The impact of health insurance status on the survival of patients with head and neck cancer. Cancer 116: 476–485. 19937673 10.1002/cncr.24774 PMC3085979

[20] Chen C-Y, Liu C-Y, Su W-C, Huang S-L, Lin K-M (2007) Factors Associated With the Diagnosis of Neurodevelopmental Disorders: A Population-Based Longitudinal Study. Pediatrics 119: e435–443. 17272605 10.1542/peds.2006-1477

[21] Liu CY Hung YT, Chuang YL, Chen YJ, Weng WS, et al (2006) Incorporating development stratification of Taiwan townships into sampling design of large scale health interview survey. J Health Manage 4: 1–22.

[22] Lin CC, Lin HC (2008) Effects of surgeon and hospital volume on 5-year survival rates following oral cancer resections: the experience of an Asian country. Surgery 143: 343–351. 18291255 10.1016/j.surg.2007.09.033

[23] Tsay JH, Lee CH, Hsu YJ, Wang PJ, Bai YM, et al (2007) Disparities in appendicitis rupture rate among mentally ill patients. BMC Public Health 7: 331. 18005406 10.1186/1471-2458-7-331 PMC2190764

[24] Kudoh A, Takase H, Takahira Y, Katagai H, Takazawa T (2003) Postoperative confusion in schizophrenic patients is affected by interleukin-6. J Clin Anesth 15: 455–462. 14652125 10.1016/j.jclinane.2003.03.008

[25] Chong LM (2001) Head and neck radiation oncology. 395–443.

[26] Kudoh A, Katagai H, Takase H, Takazawa T (2004) Effect of preoperative discontinuation of antipsychotics in schizophrenic patients on outcome during and after anaesthesia. Eur J Anaesthesiol 21: 414–416. 15141803 10.1017/s026502150422511x

[27] Schulman KA, Berlin JA, Harless W, Kerner JF, Sistrunk S, et al (1999) The Effect of Race and Sex on Physicians’ Recommendations for Cardiac Catheterization. N Engl J Med 340: 618–626. 10029647 10.1056/NEJM199902253400806

[28] Penn DL, Martin J (1998) The stigma of severe mental illness: some potential solutions for a recalcitrant problem. Psychiatr Q 69: 235–247. 9682287 10.1023/a:1022153327316

[29] Hinshaw SP, Stier A (2008) Stigma as Related to Mental Disorders. Annual Review of Clinical Psychology 4: 367–393. 10.1146/annurev.clinpsy.4.022007.141245 17716044

[30] Cooke BK, Magas LT, Virgo KS, Feinberg B, Adityanjee A, et al (2007) Appendectomy for appendicitis in patients with schizophrenia. Am J Surg 193: 41–48. 17188086 10.1016/j.amjsurg.2006.06.034

[31] Peters LJ, O’Sullivan B, Giralt J, Fitzgerald TJ, Trotti A, et al (2010) Critical impact of radiotherapy protocol compliance and quality in the treatment of advanced head and neck cancer: results from TROG 02.02. J Clin Oncol 28: 2996–3001. 20479390 10.1200/JCO.2009.27.4498

[32] Kubicek GJ, Kimler BF, Wang F, Reddy EK, Girod DA, et al (2011) Chemotherapy in head and neck cancer: clinical predictors of tolerance and outcomes. Am J Clin Oncol 34: 380–384. 20881477 10.1097/COC.0b013e3181e9c0a2

[33] Siahpush M, English D, Powles J (2006) The contribution of smoking to socioeconomic differentials in mortality: results from the Melbourne Collaborative Cohort Study, Australia. J Epidemiol Community Health 60: 1077–1079. 17108305 10.1136/jech.2005.042572 PMC2465498

